# The Broad Street pump revisited: dairy farms and an ongoing outbreak of inflammatory bowel disease in Forest, Virginia

**DOI:** 10.1186/1757-4749-3-20

**Published:** 2011-12-23

**Authors:** Ellen S Pierce, Stephen M Borowitz, Saleh A Naser

**Affiliations:** 1 13212 East Blossey Avenue, Spokane Valley, Washington, 99216 USA; 2 Division of Pediatric Gastroenterology, Hepatology and Nutrition, University of Virginia Children's Hospital, Box 800386 HSC, Charlottesville, Virginia 22908 USA; 3 Department of Molecular Biology and Microbiology, Burnett School of Biomedical Sciences, BMS Building, Room 221, University of Central Florida College of Medicine, 4000 Central Florida Boulevard, Orlando, Florida, 32816 USA

**Keywords:** inflammatory bowel disease, paratuberculosis, cluster, outbreak, aerosolization, fecal-oral waterborne transmission

## Abstract

We report an ongoing outbreak of ulcerative colitis and Crohn's disease in Forest, Virginia involving 15 unrelated children and teenagers who resided in close proximity to dairy farms. Some of our cases demonstrated serologic evidence of *Mycobacterium avium *subspecies *paratuberculosis *infection, suggesting its potential role as an etiologic agent.

## Findings

**Figure 1:**
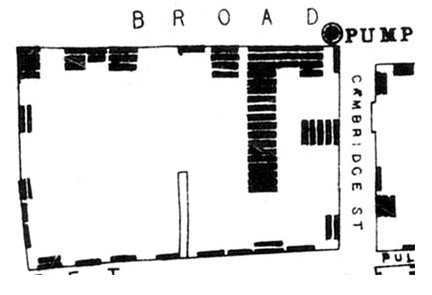
**Map of the 1854 London cholera outbreak**. The pump is indicated by a circle in the upper right hand corner of the figure. Deaths from cholera in each household are indicated by bars. Reprinted with the very kind permission of Dr. Ralph Frerichs from his John Snow website, http://www.ph.ucla.edu/epi/snow/mapsbroadstreet.html.

On December 4, 1854, at a meeting of the London Epidemiological Society, Dr. John Snow presented a map illustrating multiple cases of cholera deaths earlier that year in an area of London near a now famous pump on Broad Street (Figure 1)[1]. The word "outbreak" was used to describe the deaths from cholera occurring within houses that obtained water from the Broad Street pump, and the map of the cases' houses in relationship to the pump helped establish that an infectious microorganism in the pump's water was the cause of the cases' cholera.

Multiple cases of Crohn's disease occurring within a geographic area have been referred to as clusters rather than outbreaks, but some investigators of these clusters have concluded that some cases of Crohn's disease, like cholera, are caused by an infectious microorganism [2]; a microorganism present in unpasteurized milk [3] and cheese [4], and in animal feces that contaminate well [4,5] and river water [6,7] and lakes and ponds used for swimming and other recreational purposes [7].

*Mycobacterium avium *subspecies *paratuberculosis *(MAP), the cause of a chronic diarrheal disease in dairy cattle called Johne's ('Yo-knee's') disease, is present in an infected dairy cow's feces and milk [8], and could play an etiologic role in Crohn's clusters. Some investigators have proposed that MAP may cause some cases of Crohn's disease [2,8] and the other major form of inflammatory bowel disease ulcerative colitis [9].

While the existing medical literature suggests MAP is transmitted to humans through milk and milk products, it seems more likely that most people are exposed to the organism through water contaminated with bovine fecal matter. The symptoms of ulcerative colitis and Crohn's disease are similar to many enteric infections, and most enteric infections are transmitted via the fecal-oral route.

Adult male dairy farm workers have more than an eightfold increased risk of developing ulcerative colitis [10]; however the following is the first published cluster of inflammatory bowel disease where the affected individuals lived near dairy farms and where anti-MAP IgG antibodies were detected in a number of the patients' sera.

Our eleven children with Crohn's disease and four children with ulcerative colitis were either born in Forest or moved there before any of them developed any symptoms of inflammatory bowel disease. They ranged in age from 5 to 18 1/2 years at the onset of their symptoms and their mean age at diagnosis was 12.4 years. The first case was diagnosed in 1994 and the most recent case was diagnosed in January 2011.

**Figure 2:**
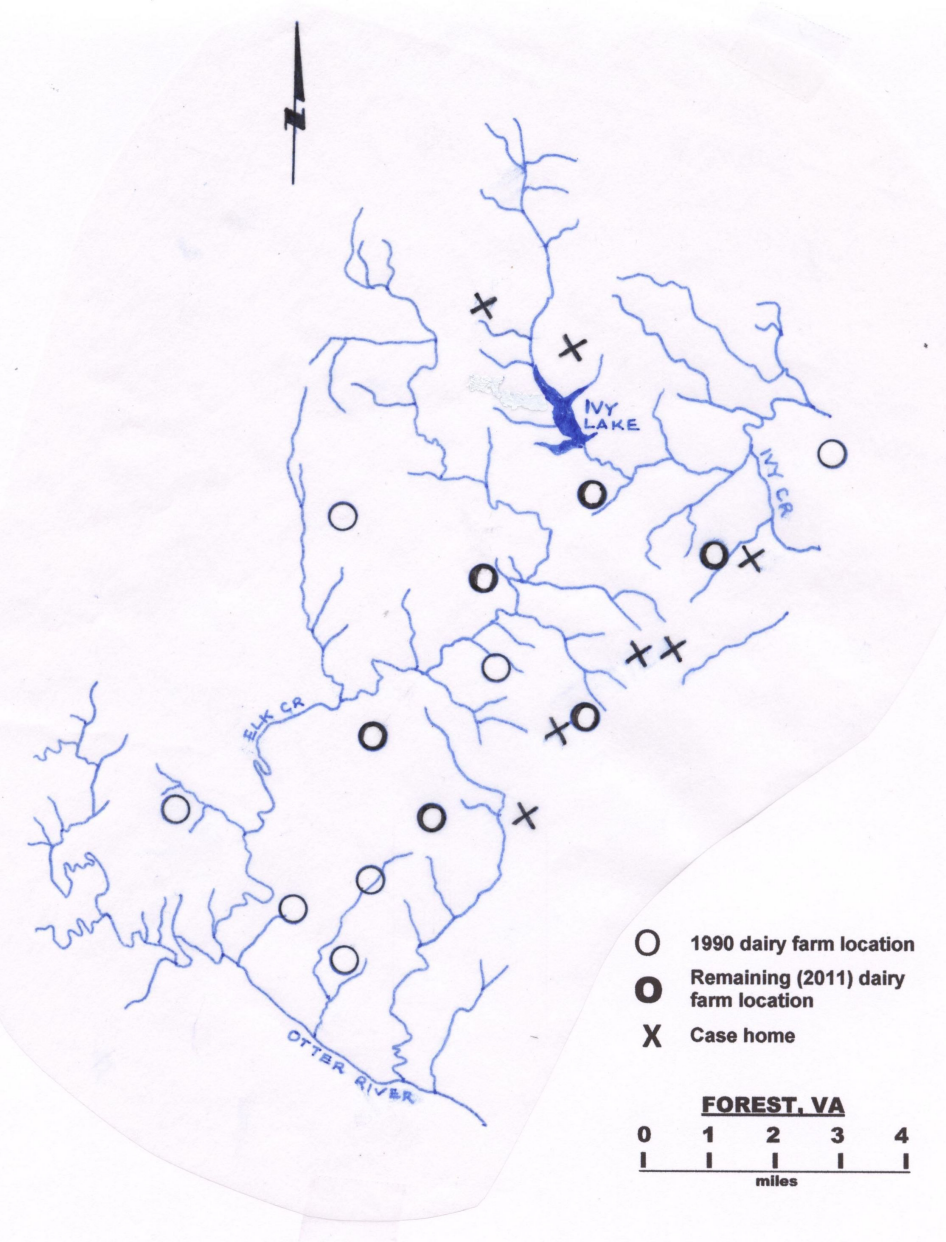
**Map of seven of the 15 cases' homes from the 1994-2011 Forest, Virginia inflammatory bowel disease outbreak**. Note that two case homes are immediately adjacent to dairy farms currently located in Forest, and the others are close to Otter River, Elk Creek or Ivy Creek tributaries.

Figure 2 is a map of Forest showing the locations of seven of our 15 cases' homes. Two homes are immediately adjacent to dairy farms and the others are close to Ivy Lake, Otter River, Elk Creek or Ivy Creek or their tributaries, all of which receive rain water runoff from four of the six farms currently located in Forest.

Blinded serum samples collected from seven of our children with Crohn's disease and two of our children with ulcerative colitis were tested for anti-MAP antibodies using p35 and p36 MAP-specific antigens. This enzyme linked immunosorbent assay is a quantitative measurement of MAP exposure [11]. Five of the seven samples from children with Crohn's disease were strongly positive for MAP antibodies.

The terms "cluster" and "outbreak" have been used to describe a time-space grouping of a condition or disease. The United States' Centers for Disease Control and Prevention differentiates between these two terms in the following manner.

An **outbreak **or an **epidemic **exists when there are *more cases of a particular disease than expected in a given area, or among a specific group of people, over a particular period of time*. An aggregation of cases...regardless of whether the number of cases is more than expected, is a **cluster **[12].

Do our 15 cases of ulcerative colitis and Crohn's disease in Forest constitute a cluster or an outbreak? The reported incidence of pediatric Crohn's disease in industrialized countries is as high as 4.56 per 100,000 children per year [13]. In the 2000 census, Forest had a population of 8006, 2369 of whom were under 18 years of age. Three of our children with Crohn's disease who tested positive for MAP were 13 year old unrelated friends who played in each others' backyards and nearby creeks and developed symptoms of inflammatory bowel disease within seven months of each other. This represents an incidence rate of 217 cases per 100,000 children per year; *more than 47 times *the expected rate. We believe an incidence rate 47 times the expected rate qualifies this cluster as an outbreak.

In 2005, S.M.B. invited representatives from the Commonwealth of Virginia's Department of Health and the Centers for Disease Control and Prevention to review his cases of Crohn's disease from Forest and surrounding areas. While the investigators concluded that a cluster of Crohn's disease was present in Forest, no further steps were taken to determine the cause or causes of the cluster. From discussions with other physicians who care for people in Forest, we estimate an additional 45 individuals who live in Forest have been diagnosed with ulcerative colitis or Crohn's disease in the past 15 years. This appears to be an ongoing outbreak with our most recent case diagnosed in January 2011 that offers a unique opportunity to investigate possible etiologic factors involved in inflammatory bowel disease, and we encourage the Commonwealth of Virginia's Department of Health and the Centers for Disease Control and Prevention to reopen their investigation into this outbreak.

While MAP can be transmitted from an infected dairy cow to an uninfected calf across the placenta in-utero or via contaminated colostrum or milk after birth, the primary route of transmission in cattle is through the direct fecal-oral route [14]; the uninfected calf grazes on fields fertilized with MAP-contaminated manure, or suckles on manure coated teats. In contrast, transmission of MAP from dairy cow manure to humans appears to be indirect, and may occur when humans swallow or inhale MAP-contaminated water.

MAP is present [15] and may be concentrated in drinking water due to the organism's resistance to chlorination and its growth in biofilms and on metallic water pipes [16]. We postulate that in our outbreak, individuals inhaled aerosolized MAP from contaminated water in the river or creek tributaries near their homes. Other clusters of inflammatory bowel disease have occurred along rivers [6,7] and near lakes [7] that may have been contaminated with dairy cow feces.

MAP infection is endemic in dairy herds in the United States [17]. Although the prevalence of MAP infection in the dairy farms located in Forest is unknown, it is well documented that MAP is present and persists for years in the environment of almost three quarters of all dairy farms [8,14,17].

Infected cows shed up to 1.6 × 10^7 ^organisms per 2 *grams *(0.07 ounces) of manure [14], a dose large enough to establish infection in a young calf, and excrete 12 to 15 *gallons *of such heavily contaminated manure per day. Liquid manure is usually collected in "lagoons" and then applied as fertilizer to agricultural land or spread onto the fields that dairy cows graze on [8,14].

Rainfall can wash MAP-contaminated manure spread on fields or crops into nearby bodies of water. MAP has an extremely hydrophobic cell wall which causes the organism to adhere to and be concentrated on the surface of bodies of water. Bursting air bubbles can then aerosolize and further concentrate [18] the organism [8]. MAP can be concentrated 10,000 fold in the aerosolized water droplets that "are small enough to enter human alveoli."[16]

Inhalation is a common route of infection by *Mycobacterium avium *complex organisms (of which MAP is a subspecies) [8], which cause gastrointestinal as well as respiratory diseases [19].

Nine of our eleven children with Crohn's disease were young males at the time of their diagnosis, in distinction to previous literature suggesting boys are perhaps twice more likely than girls to develop Crohn's disease [13]. The marked male predominance in our patients may reflect the fact that boys are more likely to play in creeks and streams than girls, but it may also be due to immunological differences between boys and girls putting boys at risk for particular infectious illnesses [20].

Other immunologic differences in our patients could put them at risk for inflammatory bowel disease including polymorphisms of the NOD-2 gene which codes for an intracellular pattern recognition molecule that is part of the innate immune response to intracellular pathogens including organisms of the genus *Mycobacterium *such as MAP [21,22]. Polymorphisms of NOD-2 are associated with Johne's disease in dairy cattle [23]. Loss of function mutations of the NOD-2 gene have been identified in approximately 15% of people affected with Crohn's disease [24] and NOD-2 deficient mice have impaired resistance to mycobacterial infections [25].

This case series adds to the circumstantial evidence that MAP may cause or at least initiate some cases of ulcerative colitis and Crohn's disease. The primary route of transmission of MAP from dairy cattle feces to humans is likely contaminated water.

## Competing interests

The authors declare that they have no competing interests. The authors received no funding for this case report.

## Authors' contributions

ESP was contacted by the parents of the seven young people whose homes are depicted in Figure 2 and wrote a first draft of the manuscript. SAN edited the manuscript and performed serum MAP testing on the nine children reported in the manuscript. SMB provided information on the additional children we discuss in the findings and extensively co-edited the manuscript. All authors have read and approved the final manuscript.
